# The Relative Contribution of Facial and Body Information to the Perception of Cuteness

**DOI:** 10.3390/bs14010068

**Published:** 2024-01-19

**Authors:** Jihyun Hwang, Yejin Lee, Sung-Ho Kim

**Affiliations:** Department of Psychology, Ewha Womans University, 52 Ewhayeodae-gil, Seodaemun-gu, Seoul 03760, Republic of Korea

**Keywords:** cuteness perception, person perception, baby schema, face and body, body proportions

## Abstract

Faces and bodies both provide cues to age and cuteness, but little work has explored their interaction in cuteness perception. This study examines the interplay of facial and bodily cues in the perception of cuteness, particularly when these cues convey conflicting age information. Participants rated the cuteness of face–body composites that combined either a child or adult face with an age-congruent or incongruent body alongside manipulations of the head-to-body height ratio (HBR). The findings from two experiments indicated that child-like facial features enhanced the perceived cuteness of adult bodies, while child-like bodily features generally had negative impacts. Furthermore, the results showed that an increased head size significantly boosted the perceived cuteness for child faces more than for adult faces. Lastly, the influence of the HBR was more pronounced when the outline of a body’s silhouette was the only available information compared to when detailed facial and bodily features were presented. This study suggests that body proportion information, derived from the body’s outline, and facial and bodily features, derived from the interior surface, are integrated to form a unitary representation of a whole person in cuteness perception. Our findings highlight the dominance of facial features over bodily information in cuteness perception, with facial attributes serving as key references for evaluating face–body relationships and body proportions. This research offers significant insights into social cognition and character design, particularly in how people perceive entities with mixed features of different social categories, underlining the importance of congruency in perceptual elements.

## 1. Introduction

We build an impression of an individual (i.e., a person percept) by referring to their physical appearance. The face and body both provide information about a person’s attributes, such as their identity, race, gender, and age, and even more psychological attributes, including their emotional states, personality traits, and attractiveness. Traditionally, research on person perception has focused on either faces or bodies alone, and despite the fact that they typically appear simultaneously, little work has explored how they are perceptually integrated into a whole person. The current study investigated the face–body interaction in the perception of cuteness when the face and body convey conflicting information about age.

### 1.1. Baby Schema and Cuteness Perception

Cuteness is a type of attractiveness associated with infantile features. Ethologist Konrad Lorenz proposed the idea that babies and children have a particular configuration of physical features, which is perceived as cute and triggers an innate releasing mechanism for care-taking and affective behaviors [1]. These features are known as the baby schema (Kindchenschema), which includes “a relatively large head, predominance of the brain capsule, large and low-lying eyes, bulging cheek region, short and thick extremities, a springy elastic consistency, and clumsy movements” [2]. In a species whose young rely on adults’ care, such a bias could have developed through biological evolution as a way to increase the chances of offspring’s survival. The behavioral effects of the baby schema have been experimentally confirmed, such that the baby schema modulates perception and attention at early stages of visual processing, enhances performance in careful behavior, and activates the brain reward system (e.g., [3,4,5,6,7,8,9]).

The majority of baby schema features are related to infant craniofacial characteristics, with infants typically possessing a predominance of the brain capsule (a large forehead), large and low-lying eyes, and a bulging cheek region. Thus, most prior research of cuteness perception has focused on those features and their configuration. Early studies, which manipulated craniofacial features of the baby schema using line drawings and schematic faces, showed that such features indeed elicit cuteness perception and caregiving responses [10,11,12,13,14,15]. More recent studies, which parametrically manipulated baby schema features using modern graphic and morphing techniques, confirmed earlier reports by showing that altering infant-like aspects (e.g., cephalic curvature) influenced the cuteness of both human and animal faces [3,4,16,17].

While these studies suggest that facial features play important roles in the perception of cuteness, another source of infantile features, which has been relatively less highlighted, is body proportions—i.e., infants’ relatively large heads, plump body shapes, and short and thick extremities [2]. The course of physical growth and development accompanies a systematic change in body proportions, that is, there is a decrease in the ratio of the head height to body height, which is due to the relatively faster growth of the legs (and the arms) than that of the head. Previous studies provided empirical evidence that the ratio of the head height to body height (HBR) can work as critical information in both age estimation and cuteness perception [11,12,18,19]. Pittenger and Todd [19] reported that the perceived ages of various agents (e.g., line drawings of human, alien, robot, and even personified flower figures) increased with a decreasing HBR. Alley [11] also reported that line drawing silhouettes of human images were perceived to be both older and less cute as the HBR decreased. Dijker et al. [20] found that a high HBR, as well as a fatty body, make adults appear more like children. In sum, the HBR affects the perception of cuteness by providing visual information about growth and age.

### 1.2. Integrating Faces and Bodies in Whole Person Perception

In the abovementioned studies, craniofacial characteristics and body proportions were investigated separately as distinct baby schema features. In the natural environment, however, a face or a body is usually encountered not as an isolated object but in the context of a whole person. Just as both faces and bodies are holistically processed, e.g., [21,22,23,24,25], a whole person is represented as a perceptual gestalt, which is qualitatively different from representing a person as the sum of their parts [26]. Lorenz [2] originally viewed the baby schema as a gestalt of infantile physical features, but it has not been tested whether the craniofacial and bodily features of the baby schema are perceived as distinct components or as an integrated, gestalt-like unit in cuteness perception.

Faces and bodies typically convey consistent and matching information about an individual’s physical and psychological conditions, e.g., [27,28,29]. However, when emotional congruency between the body and facial expressions is manipulated using face–body composite stimuli, observers’ perceptions of facial expressions are significantly influenced by the context provided by the body [30,31,32]. In Aviezer et al.’s study [31], for example, participants judged the emotions of professional tennis players in photos taken during matches. When evaluating the face–body composites or bodies alone, the winners and losers were distinguishable. However, when judging based on the faces alone, the winners’ and losers’ expressions were indistinguishable.

More related to the current study, research on attractiveness perception suggests that while face and body attractiveness are independent predictors of whole-person attractiveness [29,33], their interaction can also significantly contribute to the attractiveness of the full body [34]. Using face–body composites, Alicke et al. [34] found an interaction of face and body attractiveness, such that a minimally attractive body reduced the perception of the overall attractiveness of a whole person regardless of the level of facial attractiveness.

### 1.3. Current Study: Research Questions and Hypotheses

In the current study, we investigated the interactions of the age-related information provided by the face and body in influencing cuteness perception. For this purpose, we adopted the face–body composite paradigm; faces of two age categories (children and adults) were paired with bodies of either the same or different category, such that four types of face–body composites (adult face–adult body, adult face–baby body, baby face–adult body, and baby face–baby body) were generated. In addition, we varied the HBR by manipulating the size of the head, e.g., [35]. The participants rated the cuteness of each composite image.

In two experiments of the current study, we investigated the following three questions. First, we examined how the perceived cuteness is influenced by the interaction between facial and bodily features when they convey conflicting information about age (Experiment 1). It has been well reported in prior studies that even adults who conform to the baby schema in facial appearance are viewed as more youthful, innocent, naïve, warm, approachable, trustworthy, or likeable than those with lesser degrees of the baby schema characteristics and babyface stereotype; e.g., [36,37,38,39,40,41]. However, a discrepancy between the face age and body age, inherent in our incongruent face–body composites, would go beyond the range of normal variations, and the effect of such a large age discrepancy has hardly been tested in past research. Given the positive affective effects of facial babyishness, incongruent composites of a child face with an adult body were expected to increase cuteness, but it was also predicted that those who have an adult face with a child body would be rated low in cuteness, because adults with extreme baby-like features in their bodies could be negatively appraised [20]. Second, more importantly, we examined whether and how the HBR information interacts with facial information in cuteness perception (Experiment 1). While age-related information mainly comes from the internal facial features, the HBR information is extracted from the outline of the whole body. If these two sources of information are independently processed in cuteness perception, individuals who are more youthful in terms of body proportions would be rated as cuter irrespective of their facial information. However, if the face and body information is holistically integrated, we expected the facial information to modulate the HBR effect. Third, we investigated how different the effect of the HBR would be when the composite images were replaced by black silhouettes, where the only information available was the shape defined by the bounding contour (Experiment 2). We expected that the pattern of cuteness rating would differ between when the internal surface information is available and when it is unavailable, as most information about age comes from internal features. However, some systematic effect of the head shape difference between children and adults was expected as well, since child and adult faces systematically differ in external features like head shape as well as internal features [10].

## 2. Experiment 1

In Experiment 1, we examined (a) the effect of age congruency between the face and body and (b) the modulation of the head size effect by facial and body information in cuteness perception. The participants evaluated 108 face–body composite images in terms of perceived cuteness. Given that prior studies did not examine the cuteness perception of face–body composite images, we had participants rate each stimulus on two additional psychological attributes (likability and eeriness), as well, in order to verify whether the cuteness ratings were reliably correlated with them. 

### 2.1. Method

Participants. Thirty female undergraduates from Ewha Womans University took part in this experiment. All participants had normal or corrected-to-normal vision and received a small monetary payment in exchange for their participation. This study was approved by the university’s Institutional Review Board, and all participants provided informed consent prior to their session. One additional participant who did not complete the experiment due to computer errors was excluded from the analysis. Regarding the sample size estimation, we faced two challenges. Firstly, at the time that this study was conducted in 2016, there were no suitable analytical or simulation-based power analysis methods available for our complex linear mixed model, which included three fixed factors and their interactions. Secondly, the limited amount of prior research on the interactions of facial and bodily cues in cuteness perception made it difficult to estimate an accurate effect size. We referred to a study by Aviezer et al. [30], which used face–body composite stimuli to explore the bodily context effect on facial emotion perception and included 16 participants. To ensure adequate statistical power, we opted to recruit at least 30 participants for our experiments, which is a number we considered to be reasonably large given the study’s constraints and complexities.

Apparatus. The experiment was controlled by a program written with MATLAB (The MathWorks, Natick, MA, USA) using the Psychophysics Toolbox extensions [42,43]. The participants were seated approximately 60 cm from a 19-inch CRT monitor (1152 × 864 pixels at 85 Hz), and their responses were collected via the computer keyboard.

### 2.2. Stimuli

#### 2.2.1. Image Selection

To create face–body compound stimuli, we collected full-length, front-view photographs of three adults and three 2- to 3-year-old-looking children, all female (one Asian and two Caucasians for each group), using Google Images search. The choice to use only female images aligns with our all-female participant group, aiming to minimize potential biases or variances in perception that might arise from cross-gender evaluations. Although the precise ages of the subjects in the images were unknown, the visual differences between the two age groups were distinctly apparent. For each of these images, the head and body parts were separately cropped using Adobe Photoshop.

#### 2.2.2. Resizing Head Images

The perceived head size—a determinant of perceived cuteness—might be influenced by not only its physical area (i.e., number of image pixels), but also featural and configural information, such as the head shape, hair style, and distance between facial parts. To equalize perceived head sizes across the six face identities, in a separate, preliminary experiment, 30 Ewha undergraduates who did not participate in the main experiment performed a head-size adjustment task, which consisted of a two-step procedure. In the first step, three head images (height in visual angle: 3.26°) of each age category (children or adults) were presented side by side at the center of a white screen, and the participants adjusted the sizes of the left and right images, respectively, to match them to the size of the central image, by pressing four response keys (two keys for each image). Each key press increased or decreased the image’s height by one pixel (and the image’s width was changed proportionately). Once completing the adjustment, the participants pressed the spacebar to move onto the next trial. Three adjustment trials were given for each age category, the order of which was randomized across participants, and in each trial, a different face image was placed in the center as a reference. The adjusted values (height in pixels) of each image were averaged across the trials, and the resultant mean adjustments were taken as the perceived equal head sizes for three faces of each category. In the next step, the participants adjusted the sizes of faces across the child and adult categories. The faces resized in accordance with the perceived equal size values for each category were presented in two rows of three faces for each age group. The participants adjusted the sizes of the three images in the bottom row to match them with those of the top ones using two response keys (the sizes of the bottom three images changed simultaneously with the key presses). Two trials were given by switching the positions of the adult face and child face images, across which the adjusted values were averaged. The resultant mean adjustments were taken as the perceived equal head sizes across all six faces.

#### 2.2.3. Composing Stimuli

**Figure 1:**
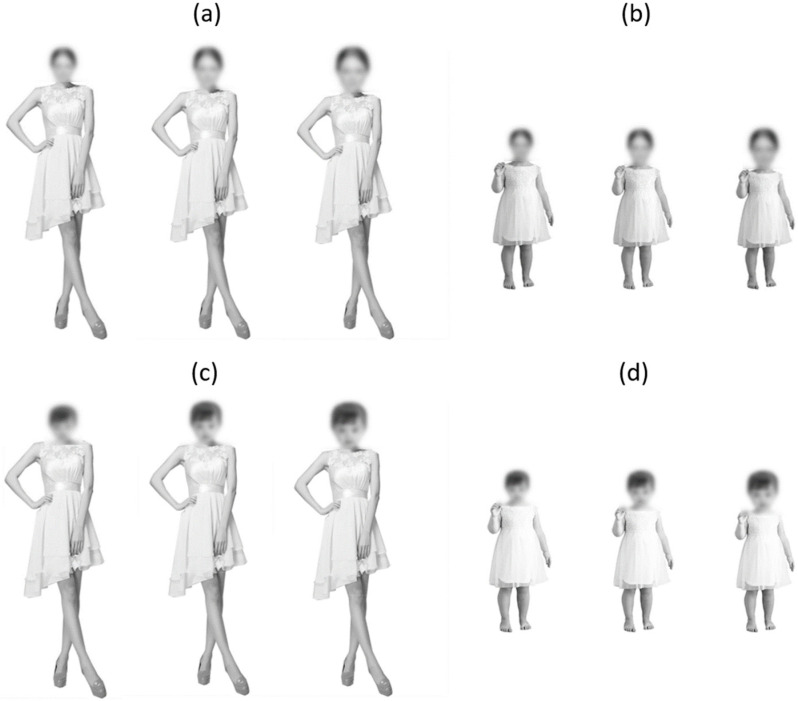
Examples of face–body composite stimuli from Experiment 1: (**a**) adult head–adult body, (**b**) adult head–child body, (**c**) child head–adult body, and (**d**) child head–child body composites with three levels of head size (small, baseline, and large from the left). Faces are blurred here for copyright issues (but not blurred in the experiment).

We generated face–body compound stimuli using a fully crossed design, such that each of the six faces was combined with each of the six bodies; thus, each face appeared once in the context of its original body and the other five times in the context of another identity’s body. The head size was manipulated in three levels (small: 85%, baseline: 100%, and large: 115%) relative to the head that was originally connected to the body; in the baseline condition, the to-be-planted heads were resized to be perceptually equal to the original head of a body; in the small or large conditions, the to-be-planted heads were resized to be 85% or 115% of the original head of a body (see Figure 1). The HBRs for the adult body and child body stimuli were, on average, 1:9.0 and 1:5.1 at the small head condition, 1:7.7 and 1:44 at the baseline condition, and 1:6.7 and 1:3.8 at the large head condition, respectively (based on the image pixel size measurement). 

To ensure that the face–body compounds looked natural, (1) we blurred the outline of the neck using the blur tool in Adobe Photoshop (blur size 19 pixels, 100% brush hardness), reducing the perceived difference in image sharpness between the body and the face around the boundary, and (2) we converted the images to gray scale, minimizing the differences in the surface properties (e.g., skin color and tone) between the face and the body. A total of 108 compound stimuli were created following a 6 face (3 children and 3 adults) × 6 body (3 children and 3 adults) × 3 head size (small, baseline, and large) factorial design, resulting in 54 age-matched (adult–adult and child–child) and 54 age-mismatched (adult–child and child–adult) head–body compounds. The height of the whole body subtended 19.89° in the images with an adult body and 11.02° in the images with a child body (i.e., 55% of the height of stimuli with an adult body), considering the average body size of two- to three-year-old children (e.g., [11]).

### 2.3. Procedure

The participants evaluated 108 face–body composite images for three attributes: cuteness, likability, and eeriness. In each trial, a composite image was presented on a white background, 8.11° to the left of the center of the screen, and remained on the screen until the participant completed all three ratings. To the right of the composite image was a 6-point Likert scale with a label for each rating task (e.g., cuteness), where “1” denoted “not cute at all” and “6” denoted “very cute” (or corresponding wording for likability and eeriness ratings). For sufficient task differentiability, the rating tasks were given sequentially, one after another; once the participants responded by pressing one of six response keys (three keys were assigned to each hand), the rating scale was replaced by another, which was again replaced by the other following the participants’ key pressing. The order of the rating tasks was counterbalanced across the participants to prevent order effects, and each participant was given a fixed order across trials.

The participants completed 12 practice trials, where face–body composite images that were not used in the experimental trials were presented, which were followed by 108 experimental trials, divided into two blocks of 54 trials each, with a short break in between. The composite stimuli were presented in a partially random sequence, such that images with either the same body or face did not appear in back-to-back trials.

### 2.4. Results

The Pearson correlation coefficients between three ratings (cuteness, likability, and eeriness, taken pairwise) were calculated for each participant. The mean correlation coefficients of the 30 participants (calculated using the Fisher z-transform) were *r* = 0.74 for a cuteness–likability correlation, *r* = −0.60 for a cuteness–eeriness correlation, and *r* = −0.81 for a likability–eeriness correlation, and one-sample *t*-test showed that all of these correlations were significant at *p*s < 0.001. These results showed that the stimuli which were rated high in cuteness were also reliably given high likability ratings, and eeriness was negatively correlated with both cuteness and likability. In the following analyses, we only considered the influence of the stimulus manipulations on the cuteness rating (see Tables S1 and S2, Figures S1 and S2 in the Supplementary Material for a summary of the results from the analysis of likability and eeriness ratings).

To analyze the effect of the congruency between the face age and body age on cuteness perception and its modulation by head size, we performed linear mixed-effects modeling (LMM) using the GAMLj module [44] in Jamovi, version 1.8.1 [45]. Unlike traditional repeated-measures ANOVAs, LMM allows us to (1) separately account for the effects caused by the experimental manipulation (fixed effects) and those that are not (random effects) and (2) control for both individual variability and the non-independence of the repeated measurements within individuals and items [46]. The fixed-effect factors included were face age (adults vs. children), body age (adults vs. children), and head size (small, baseline, and large), and all two- and three-way interactions were included, as well; the head size was polynomial-contrast-coded to test the linear and quadratic trends, and the other two factors were dummy-coded. The maximal converging random-effects structure justified by the design [47] included random intercepts for the body and face items and participants, as well as by-participant random slopes for face age and body age. The significance was calculated using the Satterthwaite’s method to estimate the degrees of freedom and generate *p* values. The full model’s specification was as follows: Cuteness ~ 1 + faceAge × bodyAge × headSize + (1 + faceAge + bodyAge|Participant) + (1|bodyNum) + (1|faceNum). 

**Figure 2:**
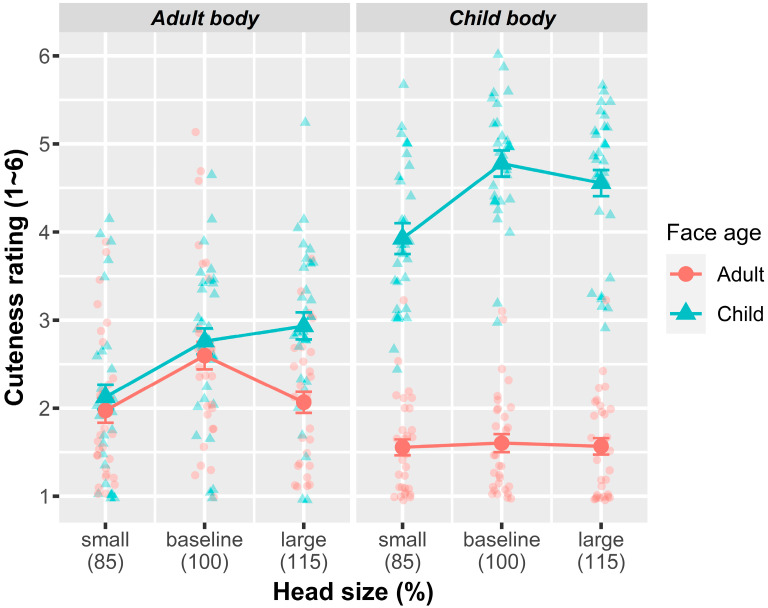
Results of Experiment 1. Mean cuteness rating as a function of face age, body age, and head size. In this and following graphs, error bars represent 95% confidence intervals, and small transparent dots represent individual participants’ mean cuteness ratings.

Figure 2 depicts the results of Experiment 1. An omnibus test of the fixed-effect terms indicated that all three fixed-effect factors disclosed the main effects (see Table 1 for model outputs): images with a child face (*M* = 3.51, *SE* = 0.22) were rated as cuter than those with an adult face( (*M* = 1.89, *SE* = 0.21), *F*(1, 6.69) = 31.31, *p* < 0.001); images with a child body (*M* = 3.00, *SE* = 0.16) were rated as cuter than those with an adult body ((*M* = 2.41, *SE* = 0.18), *F*(1, 17.46) = 23.89, *p* < 0.001); and both large (*M* = 2.78, *SE* = 0.16) and small heads (*M* = 2.40, *SE* = 0.16) lowered the cuteness rating compared with the baseline ((*M* = 2.93, *SE* = 0.16), *F*(2, 3133) = 100.45, *p* < 0.001). The polynomial contrast of the head size indicated that both the quadratic and linear trends were significant (*β* = 0.27, *SE* = 0.03, and *p* < 0.001 (linear trend) and *β* = −0.28, *SE* = 0.03, and *p* < 0.001 (quadratic trend)). 

The most critical observations for this experiment were the interactions between face age, body age, and head size. A face age × body age interaction was statistically significant (*F*(1, 3133) = 1467.73, *p* < 0.001); child faces were rated cuter when combined with a child body (*M* = 4.42, *SE* = 0.23) than with an adult body (*M* = 2.61, *SE* = 0.23), but adult faces were rated cuter when combined with an adult body (*M* = 2.21, *SE* = 0.23) than when combined with a child body (*M* = 1.58, *SE* = 0.20). Thus, the rating was higher in the order of child face–child body > child face–adult body > adult face–adult body > adult face–child body, and differences between all consecutive conditions (Bonferroni corrected) were significant at *p*s < 0.001. A face age × head size interaction was also significant (*F*(2, 3133) = 36.80, *p* < 0.001); the polynomial contrasts of head size in its interaction with face age revealed a significant interaction of the linear trend of head size (*β* = 0.47, *SE* = 0.06, *p* < 0.001), but not of the quadratic trend (*β* = −0.06, *SE* = 0.06, *p* = 0.275). A follow-up simple effect analysis showed a linear increasing trend in the head size in the cuteness rating for child faces (*β* = 0.51, *SE* = 0.04, *p* < 0.001), but not for adult faces (*β* = 0.04, *SE* = 0.04, *p* = 0.349). An interaction between body age and head size was not significant (*F*(2, 3133) = 2.76, *p* = 0.064).

Finally, two-way interaction effects were, in turn, qualified by a significant three-way interaction between face age, body age, and head size (*F*(2, 3133) = 19.38, *p* < 0.001). Notably, the interaction involving the quadratic trend of head size was significant (*β* = −0.69, *SE* = 0.11, *p* < 0.001), indicating an inverted U-shaped relationship, whereas the linear trend did not show significance (*β* = −0.07, *SE* = 0.11, *p* = 0.539). Subsequent simple interaction analyses, separated by body age, were conducted to explore the nature of this interaction involving the quadratic trend of head size. For composites with adult bodies, the quadratic trend was moderated by face age (*β* = 0.28, *SE* = 0.07, *p* < 0.001), such that the inverted U-shaped trend was more pronounced with child faces (*β* = −0.47, *SE* = 0.06, *p* < 0.001) compared to adult faces (*β* = −0.19, *SE* = 0.06, *p* = 0.002). For child body composites, the quadratic trend of head size was moderated by the face age in an opposite direction (*β* = −0.40, *SE* = 0.07, *p* < 0.001), such that a negative quadratic trend was significant when combined with a child face (*β* = −0.43, *SE* = 0.06, *p* < 0.001), but not when combined with an adult face (*β* = −0.03, *SE* = 0.06, *p* = 0.530). 

A visual inspection of Figure 2 further indicated that for adult body composites, the cuteness ratings for both adult and child face conditions followed a similar pattern of increase from small to baseline head sizes. In contrast, for the large head size, the pattern of ratings diverged, showing an increasing cuteness rating for the child face composites but a decreasing rating for the adult face composites. Simple effect analyses corroborated these observations, indicating that while the difference in cuteness ratings between adult and child face composites was not statistically significant for small (*β* = 0.15, *SE* = 0.30, *p* = 0.625) and baseline head sizes (*β* = 0.16, *SE* = 0.30, *p* = 0.60), the large head size condition yielded a significant difference, with the child face composites being rated as cuter than the adult face composites (*β* = 0.87, *SE* = 0.30, *p* = 0.021).

### 2.5. Discussion

The main results of Experiment 1 can be summarized as follows. First, composite images involving a child’s face were perceived as cuter when combined with a child body than with an adult body, but the cuteness of the adult face stimuli was much lower with a child body than with an adult body. This suggests that while child-like facial features enhance the perceived cuteness of adult bodies (i.e., babyfacedness effect), child-like bodily features have a negative impact on perceived cuteness, possibly due to negative feelings induced by a perceptual mismatch which deviates from typical developmental expectations. Second, the effect of relative head size was moderated by face age, but not by body age, such that with an increasing head size, the perceived cuteness increased for child faces more than for adult faces. This suggests that facial information provides a reference for the cuteness-related evaluation of the given HBR. Finally, the three-way interaction between the face age, body age, and head size suggests that the effect of the HBR was influenced by the congruency of face and body information, such that the inverted U-shaped effect of the head size was more prominent in the age-matched (adult–adult and child–child) than age-mismatched (adult–child and child–adult) head–body compounds. In sum, the findings of Experiment 1 suggest that body proportion information from the outline of the whole body and facial and bodily featural information are integrated to form a unitary perception of a whole person.

However, there was a possibility that internal facial information was confounded with head shape, in that adult and child faces could differ in terms of external features (e.g., head shape and hair style) as well as internal features. If there was some correlation of age-related information between internal and external facial features, the outline of the full body alone could also produce similar results. Experiment 2 tested this possibility.

## 3. Experiment 2

Experiment 1 demonstrated the influence of facial age information in the evaluation of the HBR for cuteness judgment. While facial age information is highly likely to be extracted from internal facial features (eyes, nose, and mouth) and their configuration, child and adult faces could systematically differ in external features like head shape, as well as internal features [10]. Experiment 2 thus investigated the potential contribution of the shape of the head outline in the modulation of the HBR effect on cuteness perception by using a silhouette stimulation covering the image of the person in black.

### 3.1. Method

**Figure 3:**
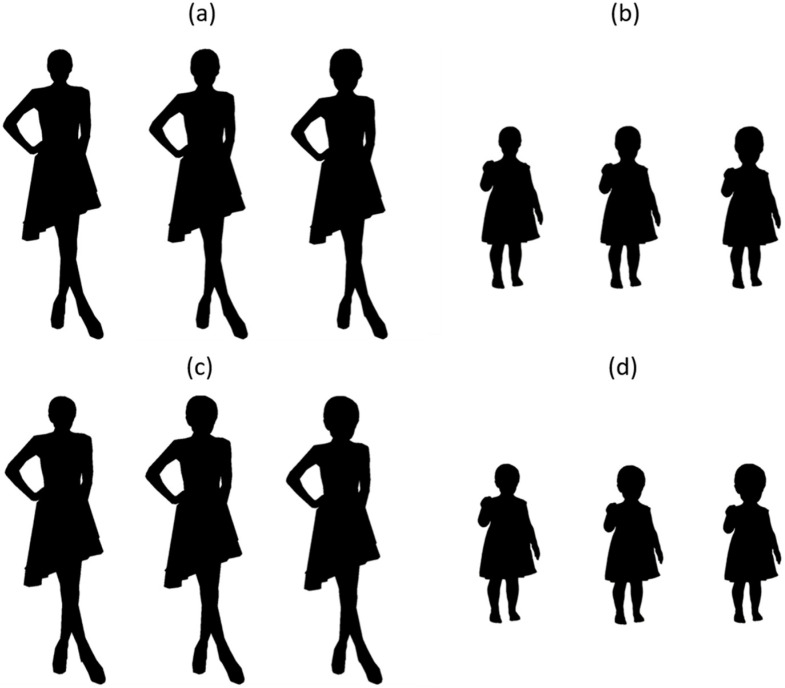
Examples of face–body composite stimuli used in Experiment 2, which were black silhouettes of those used in Experiment 1. (**a**) adult head–adult body, (**b**) adult head–child body, (**c**) child head–adult body, and (**d**) child head–child body composites with three levels of head size (small, baseline, and large from the left).

Thirty new undergraduates from Ewha Womans University, who were naive to the purpose of the experiment, participated in Experiment 2. For this experiment, the composite images from Experiment 1, were transformed into one-tone black silhouettes using Adobe Photoshop (Figure 3). The participants rated the cuteness of the silhouettes using the same procedure as in Experiment 1. However, ratings for attractiveness and eeriness were not collected in Experiment 2. All other aspects of the design and procedure were identical to those in Experiment 1.

### 3.2. Results

We conducted the same analyses as in Experiment 1 using linear mixed-effects modeling; we entered the face age, body age, and head size as fixed effects, with all two- and three-way interaction terms as well. The maximal converging random-effects structure justified by the design included random intercepts for face and body items and participants.

**Figure 4:**
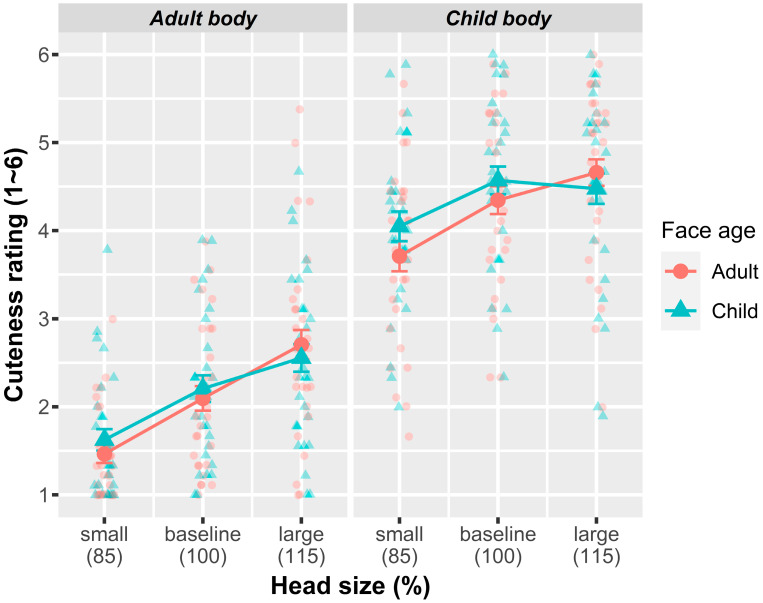
Results of Experiment 2. Mean cuteness rating as a function of face age, body age, and head size.

Figure 4 depicts the results of Experiment 2. An omnibus test of the fixed-effect terms (see Table 2 for model outputs) indicated that, unlike in Experiment 1, the composite images with a child face (*M* = 3.25, *SE* = 0.16) did not significantly differ from those with an adult face (*M* = 3.17, *SE* = 0.16) in cuteness rating (*F*(1, 4) = 1.72, *p* = 0.26). However, the main effects of the other two factors were still significant; composites with a child body (*M* = 4.30, *SE* = 0.22) were rated as cuter than those with an adult body ((*M* = 2.11, *SE* = 0.18) *F*(1, 4) = 169.12, *p* < 0.001), and perceived cuteness increased with an increasing head size (*F*(2, 2966.01) = 187.56, *p* < 0.001), with *M* = 2.72 and *SE* = 0.14 for small, *M* = 3.31 and *SE* = 0.16 for baseline, and *M* = 3.60 and *SE* = 0.18 for large head sizes. The polynomial contrast of the head size indicated that both the quadratic and linear trends were significant (*b* = 0.63, *SE* = 0.03, *p* < 0.001 (linear trend) and *b* = −0.12, *SE* = 0.03, *p* < 0.001 (quadratic trend)).

Unlike in Experiment 1, a face age × body age interaction was not statistically significant (*F*(1, 2966.02) = 1.57, *p* = 0.21), but a body age × head size interaction was significant (*F*(2, 2966.01) = 11.17, *p* < 0.001). The polynomial contrasts of head size in its interaction with body age revealed that both the linear and quadratic trends were significant (*β* = −0.28, *t*(2966.00) = −4.20, *p* < 0.001 (linear trend); *β* = −0.14, *t*(3133) = −2.13, *p* = 0.033 (quadratic trend)); a follow-up simple effect analysis showed that a linear increasing effect of the head size was larger for adult body stimuli (*β* = 0.76, *SE* = 0.05, *p* < 0.001) than for child-body stimuli (*β* = 0.49, *SE* = 0.05, *p* < 0.001). A negative quadratic trend was significant for child body stimuli (*β* = −0.19, *SE* = 0.05, *p* < 0.001) but not for adult body stimuli (*β* = −0.05, *SE* = 0.05, *p* = 0.291). Follow-up pairwise comparisons (Bonferroni corrected) further showed that for the adult body stimuli, any pairwise difference among three head sizes was significant (*p*s < 0.001), but for the child body condition, the difference between small and baseline conditions was significant (*p*s < 0.001), but the difference between baseline and large head conditions was not (*p* = 0.34).

Unexpectedly, the result revealed a significant face age × head size interaction (*F*(2, 2966.00) = 11.05, *p* < 0.001); the polynomial contrasts of head size in its interaction with face age revealed a significant interaction of the linear trend of head size (*β* = −0.29, *SE* = 0.07, *p* < 0.001), but not that of the quadratic trend (*β* = −0.10, *SE* = 0.07, *p* = 0.121). Follow-up pairwise comparisons (Bonferroni corrected) across face age categories further showed that when the head size was small, composites featuring a child face were rated as cuter than composites featuring an adult face (*p* = 0.036), but in the baseline and large head conditions, such a difference was not significant at *p*s > 0.21. Lastly, a face age × body age × head size interaction was not significant (*F*(2, 2966.01) = 0.60, *p* = 0.552). 

### 3.3. Discussion

The main effects of both body age and head size confirmed that perceived cuteness increases with a larger HBR when given body silhouettes [11,12,18,19]. Moreover, Experiment 2 revealed that the effect of the head size was modulated by both face and body outline information. First, a body age and head size interaction showed that the influence of head size was larger for adult body stimuli than for child body stimuli. It might be interpreted that for child body stimuli, the HBR was already high at a baseline head size such that an additional 15% increase did not lead to a corresponding increase in perceived cuteness; however, for adult body stimuli, due to relatively lower HBRs, an increasing head size was helpful to increase perceived cuteness. The unexpected face age × head size interaction also suggests some systematic influence of the head shape difference between child and adult face stimuli. Even though it is unclear what the cause of this interaction was, child face stimuli were perceived as cuter than adult face stimuli, at least when the head size was small. This trend was consistent with Alley’s [10] finding that baby-like head shapes can increase perceived cuteness when the size of the head is held constant. However, at baseline and large head conditions, this effect disappeared, possibly because the effect of an infantile head shape was quickly saturated with an increasing head size. In sum, Experiment 2 suggests that the effect of the HBR can be different when internal featural information is available and when it is not available.

## 4. General Discussion

Children’ faces and bodies both provide cues to their ages and cuteness. To gain an understanding of how these cues interact in cuteness perception, this study investigated the following three questions: (1) How is the perceived cuteness influenced by facial and bodily features when they convey conflicting information about age? (2) How is the effect of relative head size on cuteness perception modulated by facial and body information? (3) To what extent does the outline information of silhouette images contribute to cuteness perception? To address these questions, we presented the participants with face–body composite images, which were created by combining a face of either a child or an adult with an age-congruent or incongruent body and asked them to rate the cuteness of the images.

First, the results of Experiment 1 revealed an intriguing asymmetry in cuteness perception when combining a child’s face and body with their adult counterparts. Composite images featuring an adult body were rated as cuter when paired with a child’s face compared to when combined with an adult’s face. In contrast, composites involving a child’s body were rated as even less cute when matched with a child’s face compared to when combined with an adult’s face. Why did these two conditions lead to the opposite effects on cuteness perception even though they could be considered as equally unusual or odd in terms of a discrepancy in age between the face and body? 

One plausible explanation is that facial features might play a more prominent role in determining perceived cuteness than body features, as facial features encompass a multitude of the baby schema characteristics that are diagnostic for age estimation. The presence of child-like facial features may thus lead to an overall perception of “cuteness” that overrides any oddness associated with the adult body. In contrast, placing an adult face onto a child’s body may create a more pronounced and unsettling mismatch. Another speculation is that there could be a stronger psychological aversion to the combination of an adult face with a child’s body compared to the reverse case. It is well documented that, even in adults, facial babyishness generates positive appraisals, such as cuteness, tenderness, innocence, and kindness, e.g., [3,39,40,41]. In line with this “babyfacedness effect”, our results suggest that a child’s face on an adult body could be perceived as an exaggeration of youthful features, enhancing the perceived cuteness of adult body stimuli. This further implies that the perception of facial babyishness and the underlying caregiving mechanism may be overgeneralized and prone to misfire even when there is a substantial age discrepancy between the face and body. However, the same may not hold true for bodily babyishness, which may be subject to negative evaluation [20]. Combining a child’s body with an adult face could create a conspicuous disparity between the adult’s facial features and the child’s small body size and proportions. This combination may, therefore, evoke negative impressions or discomfort, as a child-like body deviates significantly from what is considered typical or expected for an adult individual with mature facial features in terms of physical development.

Second, more importantly, we examined how the effect of relative head size interacts with facial and bodily information in cuteness perception. While the majority of baby schema features are related to infant craniofacial features, a considerable amount of research has demonstrated that body size and proportions also provide information relating to age and cuteness [11,12,18,19]. If these two channels of information are independently processed in cuteness perception, their main effects are expected, but their interaction is not—that is, individuals who are more youthful in terms of body size and proportions should be rated as cuter irrespective of their facial information. However, we found that the effect of relative head size was moderated by face age, suggesting that the two sources of information are integrated to form a unitary representation of a whole person in cuteness perception. Moreover, the HBR information did not interact with body age, suggesting that facial features work as a more reliable reference than body features for the cuteness-related evaluation of the given HBR, possibly because bodies (i.e., body size and proportions) provide redundant information in processing the HBR. 

The three-way interaction between the face age, body age, and head size further highlights the complex interplay between facial and bodily features in the perception of cuteness. A high HBR, an indicator of youthfulness and cuteness, did not always increase the perceived cuteness. Instead, the effect of the HBR was influenced by the congruency of the face and body information, such that the inverted U-shaped effect of an increasing head size was more pronounced in age-matched than age-mismatched head–body composites. The most illustrative examples of such contextual influence were observed in composites featuring an adult body. For these adult body composites, there was a notable alignment in the pattern of increasing cuteness ratings between the adult and child face conditions as the head size ranged from small to baseline. However, a marked divergence occurred when the head size exceeded the baseline to become large: the cuteness ratings increased for composites with child faces but declined for those with adult faces. In essence, for the same adult body, a linearly increasing trend in cuteness ratings was observed with child faces, whereas an inverted U-shaped trend was more pronounced with adult faces. This pattern suggests that the range of HBR values anticipated from adult or child faces significantly influences the modulation of perceived cuteness by head size. These inherent expectations act as benchmarks, thereby determining how HBR variations are perceived in terms of cuteness. Specifically, it suggests that the expected range of HBRs for adult or child faces can limit or enhance the modulation of perceived cuteness by head size. In sum, this finding highlights the pivotal role of facial characteristics in shaping judgments related to cuteness.

Lastly, the differences in results between the two experiments highlighted the significance of evaluating the roles of different sources of perceptual information in their natural contexts. In situations where the outline of body silhouettes alone was available, body proportions had a substantial impact on perceived cuteness (as seen in Experiment 2). Conversely, when additional surface features of the face and body were available, the effect of head size on cuteness perception was less pronounced. Specifically, the cuteness ratings increased with head size from small to baseline, but no further increase in cuteness was observed when the head size grew from baseline to large (noted in Experiment 1).

The findings of our study can provide significant insights into the field of social perception and cognition, particularly in understanding how people perceive entities with mixed features, like animated characters or robots that merge child-like and adult-like elements. An illustrative example is the disconcerting effect of placing an adult face on a child’s body, which highlights the emotional impact of mismatched perceptual elements. This phenomenon can be viewed as a broader manifestation of the uncanny valley effect, a concept traditionally associated with the discomfort caused by artificial entities that resemble humans but are not entirely lifelike [48,49]. Our study advocates for broadening the discussion of the uncanny valley effect to encompass mismatches in features across various social categories, suggesting this as a fruitful avenue for research. This approach would contribute to a more comprehensive understanding of the psychological mechanisms driving the uncanny valley effect and underscores the critical role of congruency in perceptual elements in social cognition.

This research can also offer valuable insights for application in animation and character design, which are industries where the perception of cuteness plays a pivotal role. Our research underscores the importance of understanding how multiple facial and bodily cues interact to shape perceptions of cuteness, age, or even eeriness. While specific features like body proportions or the blend of child and adult characteristics significantly impact these perceptions when viewed in isolation, their effect can change considerably in a more holistic or realistic context. For instance, to achieve a cute appearance in adult characters, designers might consider reducing the HBR. However, this approach must be balanced with the realism of the face. If the character’s face is rendered or drawn with a high degree of realism, a low HBR might inadvertently induce a sense of eeriness instead of cuteness. 

## 5. Limitations and Future Research

On a final note, the current results should be interpreted with caution, given that we used only three different faces and bodies as stimuli for each age category. Furthermore, as the stimuli were exclusively female composites, it remains unclear if similar face and body cues influence cuteness perception in male figures. Another stimulus-related limitation concerns the lack of precise age information for the face stimuli. However, this study categorized age broadly as adults and children, focusing on the effect of the substantial age discrepancy between the face and body, rather than a continuous age spectrum. Given that the visual differences between the faces of children and adults were distinctly categorical, the exact age of the faces—whether 2 or 3 years old, for instance—likely does not undermine the validity and reliability of the current findings. Nonetheless, future research should explore whether the influence of age mismatch on cuteness perception is categorical or gradual. 

Secondly, the generalizability of our findings is somewhat constrained due to the exclusive testing of female participants, predominantly in their early 20s. This sampling bias arose from the predominance of females in the local participant pool. The literature suggests that there are individual and gender differences in cuteness perception, with variations in sensitivity to cuteness cues and caregiving responses [4,50,51,52,53]. For instance, prosocially oriented women exhibit greater sensitivity to cuteness than their counterparts [51], and young women of reproductive age (19–26 years) are more attuned to infant cuteness than older women and men of the same age group [52]. Despite these noted differences, we anticipate that the observed patterns of interactions between facial and bodily features in cuteness perception would remain consistent across diverse participant groups, in that variations in sensitivity levels do not necessarily imply differences in the interaction patterns. Conducting a large-scale study that includes both male and female stimuli and participants would yield a more comprehensive understanding of how facial and bodily cues interplay in the perception of cuteness. 

Lastly, the current study’s reliance on subjective ratings may raise questions about whether the observed effects of the face–body interaction are truly perceptual or reflect post-perceptual, cognitive judgments [54]. To delineate these possibilities, future research should incorporate methods that capture low-level visual processing. This could include behavioral measures, such as eye tracking [3,30,55], or objective, performance-based tasks. Such approaches will provide a more comprehensive understanding of the mechanisms underlying face–body interactions in cuteness perception.

## 6. Conclusions

In summary, the findings of the two experiments suggest that information from body proportions, extracted from the body’s outline, and facial and bodily features, derived from the interior surface, are integrated to form a unitary perception of a whole person. This aligns with perception research showing that surface features and contour information are processed to form an integrated representation of an object, e.g., [56,57]. Moreover, these findings suggest that facial cues specifying age play a more critical role than body information in determining perceived cuteness. This implies that facial characteristics can serve as a benchmark for assessing the relationship between the face and body and the proportions of the body.

## Figures and Tables

**Table 1:** Summary of the estimated mixed-effects model for effects of face age, body age, and head size on perceived cuteness in Experiment 1.

Fixed Effect	Estimate	*SE*	95% CI	df	*t*	*p*
(Intercept)	2.70	0.16	[2.40, 3.01]	9.0	17.28	<0.001
Face age (child face–adult face)	1.62	0.29	[1.05, 2.19]	6.7	5.60	<0.001
Body age (child body–adult body)	0.59	0.12	[0.35, 0.82]	17.5	4.89	<0.001
Head size (linear)	0.27	0.03	[0.22, 0.33]	3133.0	9.83	<0.001
Head size (quadratic)	−0.28	0.03	[−0.34, −0.23]	3133.0	−10.21	<0.001
Face age × Body age	2.45	0.06	[2.33, 2.58]	3133.0	38.31	<0.001
Face age × Head size (linear)	0.47	0.06	[0.36, 0.58]	3133.0	8.51	<0.001
Face age × Head size (quadratic)	−0.06	0.06	[−0.17, 0.05]	3133.0	−1.09	0.275
Body age × Head size (linear)	−0.09	0.06	[−0.20, 0.02]	3133.0	−1.66	0.098
Body age × Head size (quadratic)	0.09	0.06	[−0.02, 0.20]	3133.0	1.67	0.096
Face age × Body age × Head size (linear)	−0.07	0.11	[−0.29, 0.15]	3133.0	−0.62	0.539
Face age × Body age × Head size (quadratic)	−0.69	0.11	[−0.90, −0.47]	3133.0	−6.20	<0.001

**Table 2:** Summary of the estimated mixed-effects model for effects of face age, body age, and head size on perceived cuteness in Experiment 2.

Fixed Effect	Estimate	*SE*	95% CI	df	*t*	*p*
(Intercept)	3.21	0.15	[2.91, 3.51]	24.06	21.226	<0.001
Face age (child face–adult face)	0.08	0.06	[−0.04, 0.21]	4.00	1.311	0.260
Body age (child body–adult body)	2.19	0.17	[1.86, 2.52]	4.00	13.004	<0.001
Head size (linear)	0.63	0.03	[0.56, 0.69]	2966.01	19.029	<0.001
Head size (quadratic)	−0.12	0.03	[−0.18, −0.05]	2966.01	−3.625	<0.001
Face age × Body age	0.10	0.08	[−0.05, 0.24]	2966.02	1.255	0.210
Face age × Head size (linear)	−0.29	0.07	[−0.42, −0.16]	2966.00	−4.436	<0.001
Face age × Head size (quadratic)	−0.10	0.07	[−0.23, 0.03]	2966.01	−1.553	0.121
Body age × Head size (linear)	−0.28	0.07	[−0.41, −0.15]	2966.00	−4.204	<0.001
Body age × Head size (quadratic)	−0.14	0.07	[−0.27, −0.01]	2966.01	−2.131	0.033
Face age × Body age × Head size (linear)	−0.14	0.13	[−0.40, 0.12]	2966.00	−1.058	0.290
Face age × Body age × Head size (quadratic)	−0.03	0.13	[−0.29, 0.22]	2966.01	−0.26	0.795

## Data Availability

The data that support the findings of this study are available from the corresponding author upon request.

## Supplementary Materials

The following supporting information can be downloaded at https://www.mdpi.com/article/10.3390/bs14010068/s1, Table S1: Summary of the estimated mixed-effects model for effects of face age, body age, and head size on likability in Experiment 1; Table S2: Summary of the estimated mixed-effects model for effects of face age, body age, and head size on eeriness in Experiment 1; Figure S1: Mean likability rating as a function of face age, body age, and head size in Experiment 1; Figure S2: Mean eeriness rating as a function of face age, body age, and head size in Experiment 1.
