# Supplementary material for: The Relative Contribution of Facial and Body Information to the Perception of Cuteness
Source: Behav Sci (Basel). 2024 Jan 19;14(1):68. doi: 10.3390/bs14010068 (PMC10813407; doi:10.3390/bs14010068)
Supplement: Supplementary file 1 [file behavsci-14-00068-s001.zip › behavsci-2766053-supplementary.pdf]

Table S1. Summary of the estimated mixed-effects model for effects of face age, body age, and head size on likability in Experiment 1

Fixed effect	Estimate	SE	95% CI	df	t	p
(Intercept)	2.96	0.17	[2.64, 3.28]	10.2	17.95	< .001
Face age (child face – adult face)	0.62	0.28	[0.07, 1.17]	5.5	2.21	0.073
Body age (child body – adult body)	-0.12	0.12	[-0.36, 0.11]	8.0	-1.03	0.335
Head size (linear)	0.18	0.03	[0.12, 0.24]	3133.0	5.82	< .001
Head size (quadratic)	-0.44	0.03	[-0.50, -0.38]	3133.0	-14.19	< .001
Face age × Body age	3.84	0.07	[3.70, 3.98]	3133.0	54.13	< .001
Face age × Head size (linear)	0.48	0.06	[0.36, 0.60]	3133.0	7.78	< .001
Face age × Head size (quadratic)	0.05	0.06	[-0.07, 0.17]	3133.0	0.87	0.382
Body age × Head size (linear)	-0.03	0.06	[-0.15, 0.09]	3133.0	-0.53	0.594
Body age × Head size (quadratic)	0.30	0.06	[0.18, 0.42]	3133.0	4.84	< .001
Face age × Body age × Head size (linear)	-0.34	0.12	[-0.58, -0.10]	3133.0	-2.75	0.006
Face age × Body age × Head size (quadratic)	-0.98	0.12	[-1.23, -0.74]	3133.0	-8.01	< .001

Table S2. Summary of the estimated mixed-effects model for effects of face age, body age, and head size on eeriness in Experiment 1

Fixed effect	Estimate	SE	95% CI	df	t	p
(Intercept)	4.04	0.14	[-0.17, 0.20]	13.18	29.91	< .001
Face age (child face – adult face)	-0.34	0.20	[0.72, 0.90]	4.46	-1.71	0.156
Body age (child body – adult body)	0.01	0.09	[-0.58, -0.39]	5.02	0.15	0.889
Head size (small-baseline)	0.81	0.05	[-5.39, -5.09]	3133.00	17.36	< .001
Head size (baseline-large)	-0.49	0.05	[0.01, 0.37]	3133.00	-10.45	< .001
Face age × Body age	-5.24	0.08	[0.19, 0.55]	3133.00	-68.97	< .001
Face age × Head size (small-baseline)	0.19	0.09	[-0.64, -0.27]	3133.00	2.03	0.042
Face age × Head size (baseline-large)	0.37	0.09	[0.20, 0.57]	3133.00	4.00	< .001
Body age × Head size (small-baseline)	-0.46	0.09	[1.07, 1.80]	3133.00	-4.90	< .001
Body age × Head size (baseline-large)	0.39	0.09	[-2.00, -1.27]	3133.00	4.16	< .001
Face age × Body age × Head size (small-baseline)	1.44	0.19	[-0.17, 0.20]	3133.00	7.72	< .001
Face age × Body age × Head size (baseline-large)	-1.63	0.19	[0.72, 0.90]	3133.00	-8.78	< .001

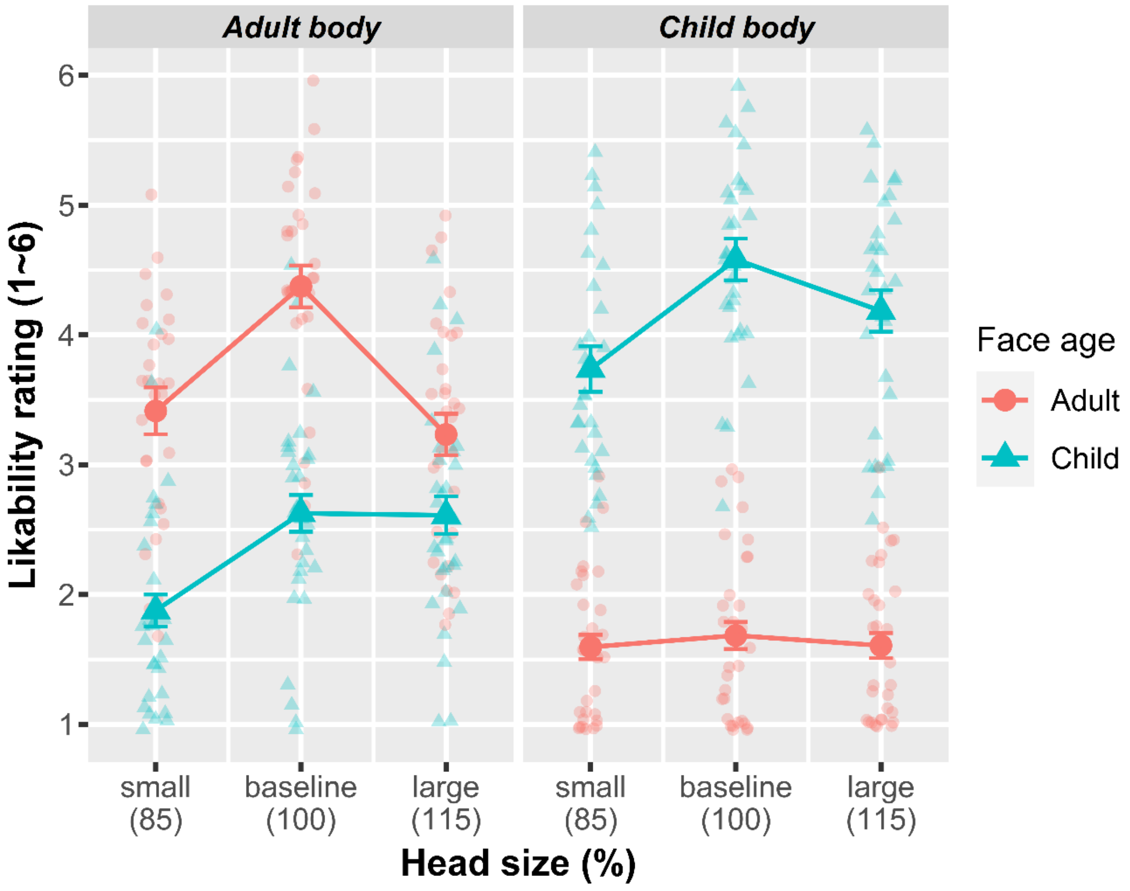

Figure S1. Mean likability rating as a function of face age, body age, and head size in Experiment 1. In this and following graphs, error bars represent 95% confidence intervals, and small transparent dots represent individual participants’ mean cuteness ratings.

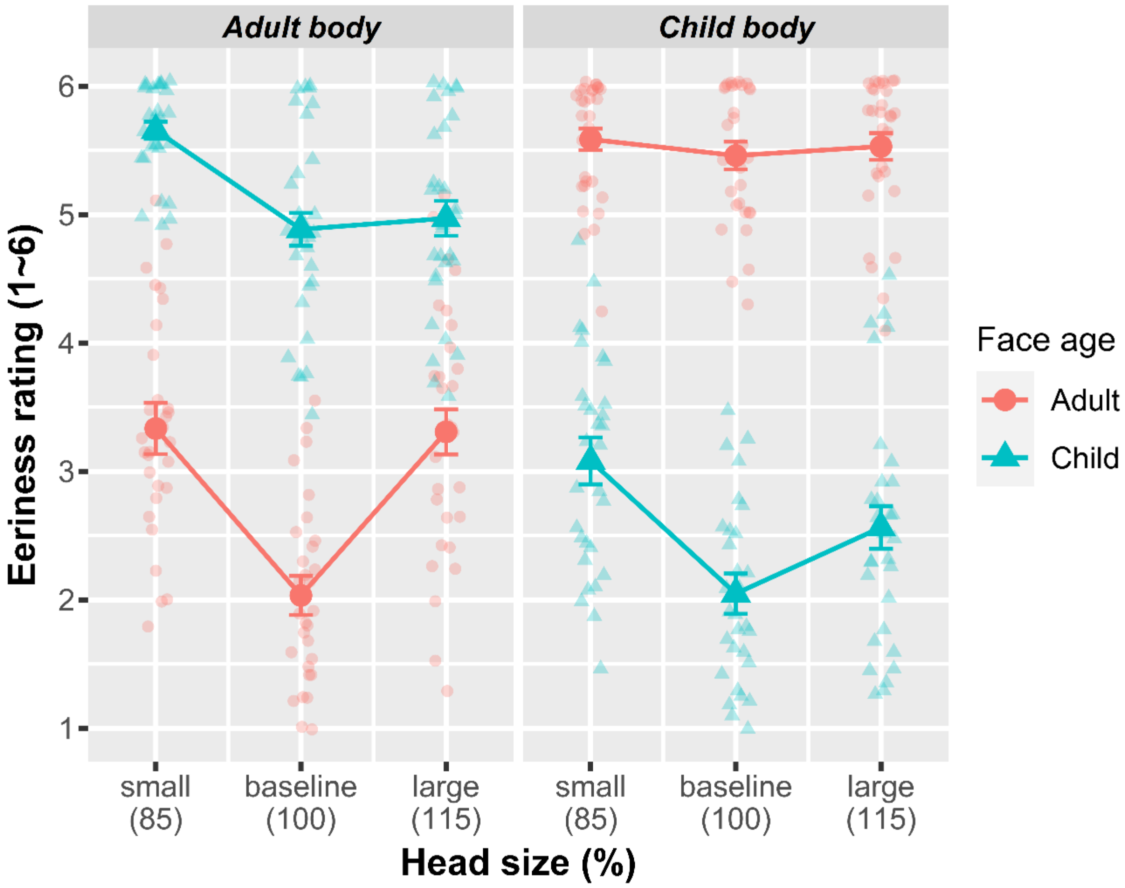

Figure S2. Mean eeriness rating as a function of face age, body age, and head size in Experiment 1.
